# Field-Applicable Recombinase Polymerase Amplification Assay for Rapid Detection of Mycoplasma capricolum subsp. capripneumoniae

**DOI:** 10.1128/JCM.00623-15

**Published:** 2015-08-18

**Authors:** Anne Liljander, Mingyan Yu, Elizabeth O'Brien, Martin Heller, Julia F. Nepper, Douglas B. Weibel, Ilona Gluecks, Mario Younan, Joachim Frey, Laurent Falquet, Joerg Jores

**Affiliations:** a International Livestock Research Institute, Nairobi, Kenya; b Friedrich-Loeffler-Institut, Federal Research Institute for Animal Health, Jena, Germany; c Department of Biochemistry, University of Wisconsin, Madison, Madison, Wisconsin, USA; d Vétérinaires Sans Frontières Suisse, Nairobi, Kenya; e Vétérinaires Sans Frontières Germany, Nairobi, Kenya; f Institute of Veterinary Bacteriology, University of Bern, Bern, Switzerland; g Biochemistry Unit, University of Fribourg and Swiss Institute of Bioinformatics, Fribourg, Switzerland

## Abstract

Contagious caprine pleuropneumonia (CCPP) is a highly contagious disease caused by Mycoplasma capricolum subsp. capripneumoniae that affects goats in Africa and Asia. Current available methods for the diagnosis of Mycoplasma infection, including cultivation, serological assays, and PCR, are time-consuming and require fully equipped stationary laboratories, which make them incompatible with testing in the resource-poor settings that are most relevant to this disease. We report a rapid, specific, and sensitive assay employing isothermal DNA amplification using recombinase polymerase amplification (RPA) for the detection of M. capricolum subsp. capripneumoniae. We developed the assay using a specific target sequence in M. capricolum subsp. capripneumoniae, as found in the genome sequence of the field strain ILRI181 and the type strain F38 and that was further evidenced in 10 field strains from different geographical regions. Detection limits corresponding to 5 × 10^3^ and 5 × 10^4^ cells/ml were obtained using genomic DNA and bacterial culture from M. capricolum subsp. capripneumoniae strain ILRI181, while no amplification was obtained from 71 related Mycoplasma isolates or from the Acholeplasma or the Pasteurella isolates, demonstrating a high degree of specificity. The assay produces a fluorescent signal within 15 to 20 min and worked well using pleural fluid obtained directly from CCPP-positive animals without prior DNA extraction. We demonstrate that the diagnosis of CCPP can be achieved, with a short sample preparation time and a simple read-out device that can be powered by a car battery, in <45 min in a simulated field setting.

## INTRODUCTION

Contagious caprine pleuropneumonia (CCPP) is one of the most severe diseases affecting goats throughout eastern Africa and many parts of Asia. CCPP is caused by Mycoplasma capricolum subsp. capripneumoniae (1), which belongs to the Mycoplasma mycoides cluster, comprising four additional closely related mycoplasmas (M. capricolum subsp. capricolum, M. mycoides subsp. *capri*, M. mycoides subsp. mycoides, and Mycoplasma leachii), all organisms which cause diseases in ruminants (2, 3). CCPP is associated with major financial losses for goat producers and imposes trade restrictions on live animals (4). The disease is characterized by fever (>41°C), coughing, and respiratory distress and is associated with morbidity and mortality approaching 90% and 60%, respectively (1). Macroscopic lesions of pleuropneumonia are often unilateral, and a very high production of pleural fluid is frequently observed postmortem (5). The clinical signs of CCPP may be confused with infections caused by other Mycoplasma spp. or Pasteurella spp. The current option for containing CCPP outbreaks is vaccination with a bacterin-type of vaccine (6). Prompt diagnosis is crucial for effective disease control and monitoring. However, the spread and burden of CCPP remain largely unknown, mainly due to inadequately funded veterinary services, an absence of infrastructure enabling swift sample transport, and the lack of rapid, inexpensive, sensitive, and specific diagnostic tests applicable for field use. Recently, a competitive enzyme-linked immunoassay (cELISA) for CCPP was modified to create a heat-stable laboratory diagnostic kit suitable for prevalence and vaccine efficacy screening; however, it is not suitable for detecting acute disease in the field (7). Thus, for diagnosing CCPP, the gold standard remains the direct isolation and cultivation of M. capricolum subsp. capripneumoniae from infected lung tissues or pleural fluid collected postmortem. However, the process is cumbersome and time-consuming due to the fastidious nature of this bacterium. In addition, bacterial isolation may be hampered by sample contamination and prior antibiotic treatment of the diseased animals. Moreover, DNA-based confirmation of the cultures using PCR methods (8–10) is still needed. Thus, culture, isolation, and molecular characterization of M. capricolum subsp. capripneumoniae is not feasible for rapid containment of a CCPP outbreak. A latex agglutination test (11) has been developed for field diagnosis of CCPP, based on the detection of serum antibodies against polysaccharide antigens. Although rapid and applicable for field use, this test is not specific for M. capricolum subsp. capripneumoniae (12). Alternatively, advanced molecular techniques, such as real-time PCR, are both sensitive and specific (10). However, methods such as these require a well-equipped laboratory, expensive equipment, and trained personnel. A loop-mediated isothermal amplification (LAMP) assay for the detection of M. capricolum subsp. capripneumoniae has been reported recently (13). This assay transcends the limitations associated with thermocyclers; however, it still requires DNA extraction protocols and gel electrophoresis to separate DNA products on horizontal agarose gels, which limits the applicability of this method as a field test. Recombinase polymerase amplification (RPA) is another isothermal technique for detecting DNA (14). RPA employs recombinase-driven oligonucleotide binding to a template sequence followed by DNA polymerase-facilitated strand extension. DNA amplification can be monitored in real time using a sequence specific fluorescent probe that fluoresces upon binding to an amplicon. This technique offers high sensitivity comparable to traditional PCR with the advantage that double-stranded DNA is exponentially amplified at constant and low temperatures (optimal temperature range, 37°C to 42°C). RPA-based diagnostic assays have been described for pathogen detection direct from biological samples, including urine samples (15). Here, we describe the development of a specific and sensitive assay using RPA for the rapid diagnosis of CCPP directly from clinical caprine specimens (i.e., pleural fluid and lung tissue samples). We demonstrate that this assay is suitable for field testing using lyophilized test reagents, a car battery for power, and a portable fluorescent illuminator to read the assay output.

## MATERIALS AND METHODS

### RPA primer and probe design.

The full genome sequence of Mycoplasma capricolum subsp. capripneumoniae strain ILRI181, isolated during a recent outbreak in Kenya (EMBL accession no. LN515399) (16), was used for primer and probe design. Oligonucleotides were designed according to the TwistDx guidelines (TwistDx, United Kingdom). The *in silico* specificities of the primers and probe were investigated using the pattern-searching tool fuzznuc from the EMBOSS package (17) against selected bacterial genomes: Mycoplasma capricolum subsp. capripneumoniae, Mycoplasma mycoides subsp. mycoides, Mycoplasma mycoides subsp. *capri*, Mycoplasma capricolum subsp. capricolum, Mycoplasma leachii, Mycoplasma putrefaciens, Mycoplasma bovis, Mycoplasma bovigenitalium, Mycoplasma arginini, Mycoplasma californicum, Mycoplasma alkalescens, Mycoplasma canis, Acholeplasma laidlawii, Pasteurella multocida, and Mannheimia haemolytica. Parameters were set to check for both the strand and the complementary strand on a circular molecule allowing 1, 5, and 10 mismatches (fuzznuc -complement -scircular -pmismatch 1, 5 or 10). The output gff file was converted to an Excel sheet by a custom script. The M. capricolum subsp. capripneumoniae-specific oligonucleotides were synthesized by Integrated DNA Technologies (USA), while the probes containing the flourophore 6-carboxyfluorescein (6-FAM) (thymidine nucleotide carrying fluorescein [dT-FAM]), a tetrahydrofuran spacer (THF), a quencher (thymidine nucleotide carrying black hole quencher 1 [dT-BHQ1]), and a 3′ block (C3-spacer) were synthesized by Biosearch Technologies, Inc. (USA).

### Mycoplasma strains and growth conditions.

For the optimization and evaluation of the RPA, M. capricolum subsp. capripneumoniae strain ILRI181 was used. Eighty-five additional strains representing 25 species and subspecies were included to investigate the specificity of the RPA (see Table S1 in the supplemental material). All strains were cultured in mycoplasma liquid medium (Mycoplasma Experience, Ltd., United Kingdom) at 37°C for 24 h to 72 h until a color change from red to orange was observed. Aliquots of M. capricolum subsp. capripneumoniae ILRI181 cells were stored at −80°C until further use in spiking experiments. Color change units per milliliter (CCU/ml) (18) were determined in duplicates for strain M. capricolum subsp. capripneumoniae ILRI181 by making 10-fold dilutions of the culture in mycoplasma liquid medium. The dilutions were grown at 37°C for 1 week prior to determining the CCU/ml.

### Isolation of genomic DNA.

Bacterial strains were grown in mycoplasma liquid medium, as described above. Cultures (10 ml) were centrifuged, the supernatants were discarded, and the cell pellets were resuspended in 500 μl of TNE buffer (10 mM Tris-HCl, pH of 8.5; 10 mM NaCl; 10 mM EDTA). Afterward, 10 μl SDS 10% and 10 μl proteinase K (20 mg/ml) was added, and the cell suspensions were incubated at 37°C for 2 h. Thereafter, 52 μl phenylmethylsulfonyl fluoride (PMSF) (100 mM in ethyl alcohol [EtOH], stored at −20°C) was added, followed by an additional incubation at room temperature for 15 min. Following the addition of 50 μl RNase A (10 mg/ml), the suspensions were incubated at 37°C for 1 h. Then 50 μl sodium acetate (1.5 M, pH of 5.2) and 550 μl buffered phenol were added; the solutions were mixed using a vortex mixer and centrifuged at 14,000 × *g* for 10 min (Centrifuge 5425, Eppendorf, Germany). The top phase was transferred to a new reaction tube; 550 μl phenol-chloroform-isoamylalcohol (25/24/1) solution was added, and the suspensions were vortexed for 20 s. Following an additional centrifugation step (14,000 × *g* for 10 min), the top phase was transferred to a new reaction tube, and the DNA was precipitated using isopropanol, washed with 70% EtOH (19), air dried, and resuspended in 100 μl Tris-HCl (10 mM, pH of 8.5). DNA concentrations (ng/μl) were determined using Nanodrop and Qubit instruments (both Thermo Fisher Scientific, Inc.).

### Clinical specimens and optimization of sample preparation.

The RPA was evaluated using a panel of pleural fluid samples (*n* = 5) and lung tissue specimens (*n* = 2) collected from goats during a recent outbreak of CCPP in Kenya in 2012 (Table 1).

**TABLE 1:** Pleural fluid and tissue samples collected during a 2012 CCPP outbreak in Kenya

Sample source	Pleural fluid		Lung tissue	
	CCU/ml	RPA	Culture	RPA
Goat 092	10^7^–10^8^	Pos^*a*^	Pos	Pos
Goat 181	10^9^	Pos	Pos	Pos
Goat DOD 7-8/10	10^8^–10^9^	Pos	ND^*b*^	ND
Goat F	10^7^–10^8^	Pos	ND	ND
Goat Y	10^9^	Pos	ND	ND

a Pos, positive.

b ND, not determined.

Tenfold dilutions in liquid medium, as described above, were used to determine the CCU/ml titer in the pleural fluid samples. Tissue samples were also cultured in liquid media for 1 week, as described above, to determine the presence of Mycoplasma isolates. As needed, the initial tissue culture was filtered through a 0.22-μm filter (Carl Roth, Germany) before diluting the filtered material 1:10 in fresh medium prior to continued growth. We evaluated different methods for sample preparation (i.e., bacterial lysis, sample dilution, and template volume) for the RPAs in duplicate on a subset of the pleural fluid samples (*n* = 2) and on available lung tissue samples (*n* = 2). Pleural fluid samples were diluted 1:20 or 1:50 in either 10 mM Tris-HCl (pH of 8.0) or nuclease-free water for subsequent heat lysis (60°C for 10 min) or in 0.2 M potassium hydroxide (KOH) or nuclease-free water for direct lysis. Five lung tissue samples from each animal were cut into small pieces (≤125 mm^3^) using a scalpel. The tissues were homogenized manually using a tissue grinder (Rotilabo; Carl Roth, Germany) in 500 μl of 10 mM Tris-HCl, 0.2 M KOH, or nuclease-free water. Samples in nuclease-free water and 0.2 M KOH were not heated, while 10 mM Tris samples were heated at 60°C for 10 min. Then, 1 or 5 μl of the different lysates (pleural fluid/lung tissue homogenate) was used as a template in a final reaction volume of 50 μl for the RPAs. After the sample preparation conditions had been optimized, the remainders of the specimens were tested in duplicate using the most suitable method. In addition to the M. capricolum subsp. capripneumoniae-positive samples described above, a panel of well-characterized M. capricolum subsp. capripneumoniae-negative archived caprine specimens, i.e., 16 lung tissue specimens and one pleural fluid sample as well as pleural fluid samples from cattle (*n* = 2) experimentally infected with Mycoplamsa mycoides subsp. mycoides (20), were included as negative clinical controls (see Data Set S2 in the supplemental material). These samples were processed using the most suitable method for sample preparation.

### Optimization of laboratory-based RPAs.

The RPA reactions were performed using the reagents and protocols from the TwistAmp exo kit (TwistDx, United Kingdom). Each 50-μl reaction mixture contained the following: 29.5 μl rehydration buffer, 12.2 or 8.2 μl nuclease-free water, 2.1 μl each of a forward primer/reverse primer (10 mM), 0.6 μl probe (10 mM), and 1.0 or 5.0 μl of template. The reagents were vortexed briefly prior to dissolving the reaction pellet. To start the reaction, 2.5 μl magnesium acetate (MgOAc, 280 mM) was added to the cap of the tube followed by a brief centrifugation (mini G; IKA, Germany), another vortex, and a final brief centrifugation. Fluorescence was measured in an ESEQuant tube scanner (Qiagen, Germany) at 42°C every 30 s for 30 min in the FAM channel (λ_excitation_ = 470 nm, λ_emission_ = 520 nm). We tested the effect of vortexing the assay 4 to 6 min after it was initiated. To distinguish positive from negative results, a cutoff value was calculated for every individual sample according to the guidelines on threshold validation in the Twista Studio software manual. A sample was deemed positive if all replicates were above three and a half standard deviations (3.5 SD) of the background during a defined time range (i.e., after 19 to 20 min of amplification). A threshold time range of 0 to 4 min 30 s was used.

### Optimization of field-applicable RPAs.

RPA reaction pellets (TwistAmp exo kit) were dissolved in buffer, primer, probe, and water, as described above but excluding template (5.0 μl) and MgOAc. Then, the RPA reactions were frozen at −80°C prior to lyophilisation in LyoQuest (Telstar life science solutions) at a temperature of −55°C and a pressure of 0.400 mBar. Aliquots of MgOAc (2.5 μl) were also lyophilized in separate tubes. Aliquots of lyophilized RPA reactions and MgOAc were either used directly or stored at 4°C and 37°C for 5 days prior to use. In addition, a subset of the lyophilized reactions was stored at 4°C for 3 months before use. To set up the reactions, 5 μl template and 45 μl of nuclease-free water was added to the lyophilized MgOAc, and this mixture was used to dissolve the lyophilized RPA reaction pellets. Immediately after dissolving (i.e., *t* = 0 min), we performed a fluorescence measurement using the tube scanner. The sensitivities of the lyophilized reactions were determined, as described below, using cultured M. capricolum subsp. capripneumoniae ILRI181 in spiking experiments. The read-out system (ESEQuant tube scanner) was tested for field applicability by running the reactions in a car using a DC-to-DC converter (120-W laptop car power supply [L40BB]) connected to a 12-V plug.

### Determination of specificities of the M. capricolum subsp. capripneumoniae primers/probe.

The specificities of the M. capricolum subsp. capripneumoniae primers and probe were determined by performing duplicate RPA reactions using genomic DNA (corresponding to 10^7^ copies of the genome) from a total of 86 bacterial strains (see Table S1 in the supplemental material). Positive controls using 10^7^ genome copies of M. capricolum subsp. capripneumoniae ILRI181 DNA were included in each run.

### Determination of RPA sensitivity.

The sensitivity of the RPA was determined using genomic DNA (corresponding to 10^0^ to 10^6^ genome copies/μl) and lysed bacteria (corresponding to 10^0^ to 10^5^ CCU/ml) of M. capricolum subsp. capripneumoniae ILRI181 in spiking experiments. Every run was repeated 8 times. The DNA was diluted 10-fold in nuclease-free water, and bacteria were diluted 10-fold in plasma (resembling pleural fluid) isolated from a healthy goat prior to a 100-fold dilution in nuclease-free water for direct lysis. Five μl of template DNA was used from each dilution in a total RPA reaction volume of 50 μl. Nuclease-free water and plasma diluted 1:100 in nuclease-free water were included in every run as negative controls for the DNA and spiking experiments, respectively.

### Assessment of RPA performance using pleural fluid and lung tissue specimens.

Pleural fluid samples (*n* = 6) were diluted 1:50 in nuclease-free water, and 5 μl of this dilution was used as the template for the RPA amplification. Lung tissue specimens (*n* = 18) were homogenized manually in 500 μl of 0.2 M KOH. One μl of the tissue homogenate was used as a template in a final reaction volume of 50 μl for the RPAs. These reactions were performed in duplicate.

## RESULTS

### *In silico* specificities of primers and probe for RPA.

The primers were designed to amplify a 245-bp region within a single-copy gene in the genome of M. capricolum subsp. capripneumoniae strain ILRI181 (locus tag MCCPILRI181_00726) (16) (Table 2). To ensure that the target sequences were unique to M. capricolum subsp. capripneumoniae, we screened the selected primers and probe *in silico* against the genomes of 14 bacteria causing infections in ruminants (see Data Set S1 in the supplemental material). We were unable to find complementary regions when allowing 1 or 5 sequence mismatches for the primer sequences. Permitting 10 mismatches did give rise to sequence matches for one of the two primers. According to this *in silico* analysis, the primers fulfilled the requirements to ensure specificity of the RPA technology (21).

**TABLE 2:** Oligonucleotide primers and probe used in this study

Oligonucleotide^*a*^	Sequence (5′ to 3′)
Mccp_F	AATCGGTTTATCAAGCCATTCGACATTCTATAAAAT
Mccp_R	GAAAATTAAACTTTGAAAGAAATAGAATTTAGTTT
Mccp_P	CTCTCTTTTATCACTAACAAAATTCAAAAAGA[dT-FAM][THF][dT-BHQ1]CCTTTAAGTCATAAAA[3′-block]^*b*^

a F, forward primer; R, reverse primer; P, probe.

b dT-FAM, thymidine nucleotide carrying fluorescein; THF, tetrahydrofuran spacer; dT-BHQ1, thymidine nucleotide carrying black hole quencher 1.

### Sample preparation optimization.

We evaluated various methods for sample preparation for the RPAs. Direct lysis of pleural fluid specimens (*n* = 2) in nuclease-free water was sufficient for release of nucleic acids from mycoplasma. We did not find a significant difference in signal intensity and onset of the RPA reaction between samples lysed in a 1:20 or 1:50 dilution in H_2_O, Tris-HCl, or KOH. Heat lysis of bacteria at 60°C for 10 min in nuclease-free water or Tris-HCl buffer (10 mM, pH of 8.5) and alkaline lysis in 0.2 M KOH reduced fluorescent signal intensities (mV) and delayed the onset of the reaction (data not shown). Subsequently, RPA reactions with pleural fluid samples were performed using direct lysis in nuclease-free water in a dilution of 1:50. Similar methods of bacterial lysis were also evaluated for lung tissue samples (*n* = 2). We tested five lung tissue specimens from each animal and found considerable variability in detecting Mycoplasma isolates in the tissue specimens used. All five specimens produced a positive M. capricolum subsp. capripneumoniae result; however, there were differences in signal intensity. Positive results were obtained within 10 to 14 min when bacterial lysis was performed using 0.2 M KOH and 1 μl lysate (see Fig. S1 in the supplemental material). Similar results were obtained when 5 μl of sample was used; the concentration of KOH had a negative impact on the appearance of fluorescence in the assay (data not shown). Direct lysis of bacteria in nuclease-free water and heat lysis in Tris-HCl buffer did not improve the signal intensity.

### Specificity of the RPA reaction.

To assess the specificity of the M. capricolum subsp. capripneumoniae RPA reaction, genomic DNA samples from a panel of Mycoplasma spp. and non-Mycoplasma spp. ruminant bacterial pathogens were tested (see Table S1 in the supplemental material). As expected, amplification was obtained only when template DNA from M. capricolum subsp. capripneumoniae strains was used. All 14 M. capricolum subsp. capripneumoniae strains tested produced a positive assay signal. All of the 71 non-M. capricolum subsp. capripneumoniae strains, as well as the negative controls, remained negative throughout the 30-min analysis period.

### Molecular sensitivity of the RPA reaction.

**FIG 1:**
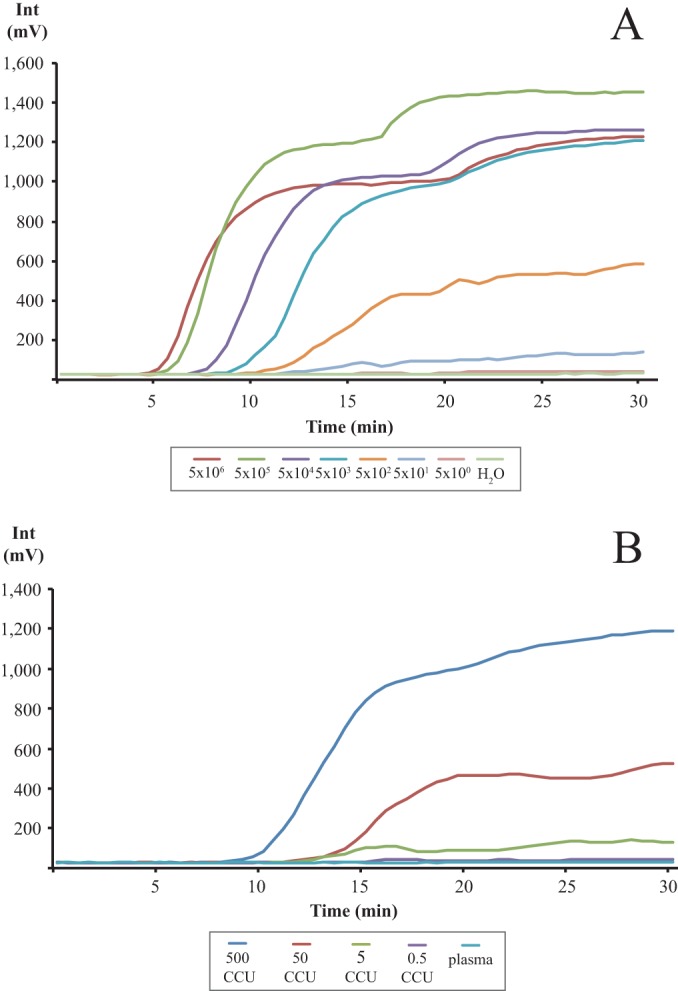
Graph depicting the RPA amplification (development of fluorescence, mV) over time (minutes). (A) M. capricolum subsp. capripneumoniae DNA (5 × 10^6^ to 5 × 10^0^ copies/reaction) diluted in nuclease-free water. (B) M. capricolum subsp. capripneumoniae CCU (500 to 0.5 CCU/reaction) spiked in plasma from a healthy goat. Each graph represents the mean value from eight individual runs.

The detection limit of the RPA reaction was assessed using 10-fold dilutions of genomic DNA extracted from M. capricolum subsp. capripneumoniae ILRI181. Eight reactions were performed for each DNA dilution. An example of the output is shown in Fig. 1A. We detected high DNA concentrations (5 × 10^6^ to 5 × 10^5^ genome copies/reaction) within 7 min of amplification and reproducibly detected 50 to 500 genome copies/reaction within 15 min. We determined the overall assay sensitivity by assaying 10-fold dilutions of cultured M. capricolum subsp. capripneumoniae strain ILRI181 diluted in goat plasma. Figure 1B depicts a representative example of the assay output. At high bacterial concentrations (i.e., 500 CCU/reaction), we detected an increase in the fluorescent signals (mV) within 10 min of amplification, while concentrations of 50 CCU/reaction were detected within 15 min. Bacterial concentrations of 5 CCU/reaction were consistently detected, albeit at low signal intensities. An additional mixing of the reaction by including a short vortexing step after 4 to 6 min did not improve the sensitivity or signal intensity (data not shown).

### RPA performance using clinical specimens from a recent Kenyan CCPP outbreak.

**FIG 2:**
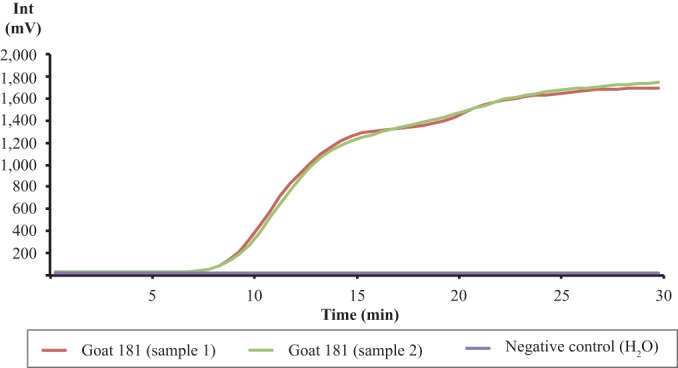
Graph depicting the amplification from pleural fluid samples from a CCPP-infected animal (Goat 181) in duplicate. Negative control, nuclease-free water.

We evaluated the RPA using pleural fluid samples (*n* = 5) and lung tissue samples (*n* = 2) collected during a CCPP outbreak in Kenya in 2012. All clinical specimens were cultured to determine presence of Mycoplasma isolates and/or CCU/ml titers. All but one replicate of the tissue cultures contained detectable Mycoplasma isolates. Bacterial titers ranging between 10^7^ to 10^9^ CCU/ml were obtained from pleural fluid samples cultured in duplicate (Table 1). We found that all pleural fluid samples produced positive assay results within 10 to 13 min after initiating the RPA reaction. An example of a sample curve from a pleural fluid sample is depicted in Fig. 2. (See Fig. S1 in the supplemental material for examples of the data from the lung tissues.) All included archived M. capricolum subsp. capripneumoniae-negative clinical specimens were negative in the RPA (see Data Set S2 in the supplemental material).

### RPA performance in a simulated field setting.

To test the storage conditions for the RPA reagents for field use, we lyophilized the RPA reaction components and MgOAc and stored them at 4°C and 37°C. Immediately prior to performing the RPAs, we dissolved lyophilized MgOAc pellets (2.5 μl) in a solution consisting of 5 μl template (cultured M. capricolum subsp. capripneumoniae) in 45 μl of nuclease-free water, and we added this solution to the lyophilized RPA reactions. The results indicated that 37°C is not a suitable temperature for storing lyophilized RPA reactions and MgOAc. We were not able to detect fluorescence signals using lyophilized RPA reagents stored at 37°C for 5 days, even when using fresh solutions of MgOAc. We were also unable to detect positive assays when we used freshly prepared solutions of RPA reagents and lyophilized MgOAc stored at 37°C. We found that lyophilized RPA reactions and MgOAc can be stored at 4°C for 5 days with acceptable compromise of the reaction signals compared with fresh RPA reactions. We determined the reaction sensitivity for lyophilized RPA and MgOAc stored at 4°C by culturing M. capricolum subsp. capripneumoniae ILRI181 and spiking the bacterium into experiments. We found a detection limit of 50 CCU/reaction in triplicate runs, which is ∼10-fold less than that in assays using fresh RPA reactions. The same results were obtained after storing lyophilized RPA and MgOAc pellets at 4°C for 3 months. A detection system based on an ESEQuant tube scanner powered using a 120-W laptop car power supply (L40BB) connected to a car cigarette lighter worked well for measuring the assay output (see Fig. S2 in the supplemental material).

## DISCUSSION

Successful surveillance and control of livestock diseases requires specific, sensitive, and rapid diagnosis of pathogens. In developed countries, laboratory-based tests, such as ELISA and real-time PCR, are useful for making rapid diagnoses, in part due to widespread clinical labs, networks of diagnostic laboratories, and courier systems that guarantee rapid transport of specimens to the laboratory. The low prevalence of such laboratories and the absence of a similar network in many regions of sub-Saharan Africa limit the feasibility of these clinical tests. Furthermore, infrastructure limitations (e.g., lack of reliable power and refrigeration) complicate the development of field-adapted assays. In this study, we developed a new diagnostic assay based on RPA and assessed its applicability to rapidly and robustly diagnose CCPP, one of the major diseases affecting small ruminants in developing countries in Africa and Asia.

The assay detected the pathogen Mycoplasma capricolum subsp. capripneumoniae specifically and sensitively from laboratory samples (purified genomic DNA), cultured bacteria, and field samples (pleural fluid from a CCPP outbreak). We demonstrated the ability to correctly differentiate between M. capricolum subsp. capripneumoniae and other closely related mycoplasmas and bacterial pathogens causing CCPP-like symptoms, which is a critical step for this type of diagnostic assay. We achieved assay specificity by engineering the RPA to reduce off-target binding and cross-reactivity with other closely related Mycoplasma strains. No amplification was obtained from a large panel of DNA samples from closely related Mycoplasma strains and other bacteria causing infections in ruminants. In contrast, we were able to correctly identify DNA samples from 14 M. capricolum subsp. capripneumoniae strains using the assay, thereby demonstrating a high degree of specificity. Amplification was detected within several minutes when using DNA extracted by conventional methods, and with as few as 50 genome copies. These results highlight RPA for diagnostic detection of M. capricolum subsp. capripneumoniae in extracted DNA. To circumvent the need for nucleic acid purification under field conditions, we evaluated several simple methods for sample preparation, including direct bacterial lysis with nuclease-free water, heat lysis, and alkaline lysis (0.2 M KOH). The heat and alkaline lyses alternatives did not result in sufficient recovery of DNA from cultured material and pleural fluid samples, as demonstrated by a delay and a low level of fluorescence produced (data not shown). We found that direct bacterial lysis with nuclease-free water worked well. Similar sensitivities were obtained from plasma samples spiked with cultured M. capricolum subsp. capripneumoniae compared to extracted DNA (Fig. 1B).

CCPP is known for its very high morbidity and high mortality and for creating pathomorphologic lesions exhibiting effusions with high concentrations of Mycoplasma isolates (in this case, we evaluated 10^7^ to 10^9^ CCU/ml). Direct isolation and cultivation of the causative bacteria from infected lung tissue samples or pleural fluid samples taken postmortem are still the gold standard for the diagnosis of CCPP. However, cultivation has limitations that are exacerbated in resource-limited areas: it requires specialized nutrient media, takes several days, and is often unsuccessful due to the fastidious nature of the organism. Currently available molecular diagnostic tools employing PCR and LAMP require either a well-equipped laboratory or extensive sample preparation, thus limiting their application in the field. We have transcended the limits of these assays and tests using an RPA-based field applicable assay to detect M. capricolum subsp. capripneumoniae directly from pleural fluid samples collected from infected animals. After diluting the sample in nuclease-free water, we were able to detect bacteria within 15 min (Fig. 2). Using this approach, a definitive diagnosis of CCPP presence in a herd can be achieved in <45 min.

In addition, we tested suitability for field applications of the method by lyophilizing the RPA reactions and MgOAc and storing them at 4°C and 37°C. The fresh in-house lyophilized RPA reagents had similar sensitivity as the fresh RPA reactions. However, after storage at 37°C for a few days, the in-house lyophilized reagents lost activity completely. The lyophilized RPA reagents can be stored at 4°C for 3 months and still maintain a detection limit of 50 CCU/reaction. The pleural fluid from CCPP-positive animals has a range of 10^7^ to 10^9^ CCU/ml; therefore, the lyophilized RPA reactions should be able to detect M. capricolum subsp. capripneumoniae from pleural fluid. The ESEQuant tube scanner is portable and can be powered by a car battery. This equipment has been distributed to 35 African countries and has been tested in their respective laboratories (22). Thus, the method described is suitable for rapid CCPP diagnosis in the field. The RPA reagents can be lyophilized in the lab and transported to the field on an ice pack, the reaction can be performed in a car, and the assay produces definitive results in <45 min. Our results indicate that RPAs are robust, rapid, specific, sensitive, and suitable for field applications. The use of other goat specimens, such as broncholavage and nasal swabs, remains to be evaluated and optimized.

The assay we describe is based on a single-copy target region in M. capricolum subsp. capripneumoniae. Ruminant Mycoplasma strains have been reported to have minimal genomes that are able to exchange DNA (23) through a conjugation-like process driven by integrative conjugative elements (24). Therefore, it would be advisable to have multiple target regions to increase the specificity of the assay. Additionally, the use of multicopy targets, if present, should be considered in the future to increase the sensitivity.

The low temperature required for RPA makes this CCPP assay an excellent foundation for the development of a novel field-applicable diagnostic tool in which the assays are integrated into a microfluidics platform and combined with a mobile phone-powered device which provides the temperature control for the assay and measures assay florescence. We are currently working on a related diagnostic platform. Importantly, this class of mobile assays that integrate mechanisms of data transmission can form the backbone of a national early warning system for disease, in which test data are disseminated through mobile phone apps.

In summary, this is the first description of an RPA-based field applicable diagnostic assay for a disease caused by Mycoplasma spp. Although this assay in moderately expensive, this technology offers significant advantages regarding speed and the potential for conversion into a next-generation diagnostic assay that is connected to mobile phone technology in resource-poor settings, such as sub-Saharan Africa and Asia.

## Supplementary Material

Supplemental material
