# Supplementary material for: Field-Applicable Recombinase Polymerase Amplification Assay for Rapid Detection of Mycoplasma capricolum subsp. capripneumoniae
Source: J Clin Microbiol. 2015 Aug 18;53(9):2810–5. doi: 10.1128/JCM.00623-15 (PMC4540935; doi:10.1128/JCM.00623-15)
Supplement: Supplemental material [file supp_53_9_2810__index.html]

Field-Applicable Recombinase Polymerase Amplification Assay for Rapid Detection of Mycoplasma capricolum subsp. capripneumoniae — Supplemental material 

# Field-Applicable Recombinase Polymerase Amplification Assay for Rapid Detection of Mycoplasma capricolum subsp. capripneumoniae

## Supplemental material

- Supplemental file 1 -

  Table S1 (Strains used in this study) and Fig. S1 (Results of RPA assay from a tissue sample) and S2 (Equipment used to run recombinase polymerase amplification method, powered by car battery)

  PDF, 2.3M
- Supplemental file 2 -

  Data Set S1 (Results of bioinformatics analysis for testing specificity of RPA primers and probe against 14 bacterial genomes of ruminant pathogens)

  XLSX, 45K
- Supplemental file 3 -

  Data Set S2 (Results of RPA runs on archived negative clinical control samples)

  XLSX, 41K
