# Supplementary material for: Field-Applicable Recombinase Polymerase Amplification Assay for Rapid Detection of Mycoplasma capricolum subsp. capripneumoniae
Source: J Clin Microbiol. 2015 Aug 18;53(9):2810–5. doi: 10.1128/JCM.00623-15 (PMC4540935; doi:10.1128/JCM.00623-15)
Supplement: Supplemental material [file JCM.00623-15_zjm999094439so1.pdf]

TABLE S1: Strains used in this study

Species	Strain designation	Country of origin	Year of isolation	Host species	Reference	Provider of strain or DNA for this study
Mycoplasma capricolum subsp. *capripneumoniae*	ILRI181	Kenya	2012	Goat	(1)	Anne Liljander
Mccp	GL97P	Tunisia	1980	Goat	(2)	Martin Heller
Mccp	87F05	Turkey	2005	Goat	(2)	Martin Heller
Mccp	F38	Kenya	1976	Goat	(1, 3)	Martin Heller
Mccp	95043	Niger	1995	Goat	(4)	Joachim Frey
Mccp	91106/550/1	Dubai	1991	Goat	(4)	Joachim Frey
Mccp	M85/98	Tanzania	1998	Goat	(5)	Joachim Frey
Mccp	Gabés	Tunisia	1980	Goat	(6)	Joachim Frey
Mccp	M79/93	Uganda	1993	Goat	(7)	Joachim Frey
Mccp	4/2LC	Oman	1988	Goat	(8)	Joachim Frey
Mccp	9231-Abomaso	Ethiopia	1982	Goat	(9)	Joachim Frey

TABLE S1: Strains used in this study

Mccp	8789	Chad	1987	Goat	(10)	Joachim Frey
Mccp/ M. leachii mixed infection	D1800/04-2	Dubai	2004	Gazelle	This study	Martin Heller
Mccp/ M. ovipneumoniae mixed infection	3535	Qatar	2005	Mouflon	This study	Martin Heller
Mycoplasma capricolum subsp. *capricolum*	California kid [ATCC27343]	USA	1955	Goat	(11)	Martin Heller
Mcc	C47	Germany	<1992	Sheep	(2)	Martin Heller
Mcc	7714	France	1967	Goat	(12)	Martin Heller
Mcc	4146	France	1997	Goat	(13)	Martin Heller
Mcc	14DD0024	Germany	2015	Human	This study	Martin Heller
Mcc	6443-90	France	1990	Goat	(14)	Martin Heller
Mcc	8086-1	France	1980	Goat	(14)	Martin Heller
Mycoplasma mycoides subsp. *capri*	153/93	Canary Islands	1993	Goat	(15)	Martin Heller
Mmc	95010-C1	France	1995	Goat	(16, 17)	Martin Heller

TABLE S1: Strains used in this study

Mmc	Wi18079	Germany	2009	Goat	(2)	Martin Heller
Mmc	D2482	Switzerland	1991	Goat	(18)	Martin Heller
Mmc	Y-goat	Australia	1956	Goat	(19)	Martin Heller
Mmc	CapriL	France	1975	Goat	(18)	Martin Heller
Mmc	PG3	Turkey	1950	Goat	(20)	Martin Heller
Mmc	My-325	Croatia	1986	Goat	(2)	Martin Heller
Mmc	G1313.94	Germany	1994	Barbary sheep	(2)	Martin Heller
Mmc	G1255.94	Germany	1994	Barbary sheep	(2)	Martin Heller
Mmc	7302	Portugal	<1994	Goat	(21)	Martin Heller
Mmc	7730	France	1994	Goat	(22)	Martin Heller
Mmc	G1283.94	Germany	1994	Barbary Sheep	(2)	Martin Heller
Mmc	80/93	Spain	1994	Goat	(2)	Martin Heller
Mmc	GM12	USA	1979	Goat	(23)	Joerg Jores

TABLE S1: Strains used in this study

Mmc	Kombolcha	Ethiopia	1975	Goat	(17)	Martin Heller
Mmc	9096-C9415	Nigeria	unknown	Goat	(24)	Joachim Frey
Mycoplasma mycoides subsp. *mycoides*	L2	Italy	1993	Cattle	(25)	Martin Heller
Mmm	PG1	Africa	1931	Cattle	(26)	Martin Heller
Mmm	TAN 8	Tanzania	1996	Cattle	(27)	Martin Heller
Mmm	Matapi	Namibia	2004	Cattle	(28)	Martin Heller
Mmm	PO2	France	1980	Cattle	(25)	Martin Heller
Mmm	Mandigwan	Namibia	2001	Cattle	(2)	Martin Heller
Mmm	Fatick	Senegal	1968	Cattle	(25)	Martin Heller
Mmm	C11	Chad	1962	Cattle	(25)	Martin Heller
Mmm	B66	Kenya	2000	Cattle	(29)	Joerg Jores
Mmm	Afadé	Cameroon	1968	Cattle	(25)	Joerg Jores
Mmm	Gladysdale	Australia	1953	Cattle	(30)	Martin Heller
Mycoplasma serogroup	B144P	USA	1956	Cattle	(31)	Joachim Frey

TABLE S1: Strains used in this study

L						
M. leachii	FRD424	India	1993	Goat	(18)	Martin Heller
M. leachii	PAD3186	India	1993	Goat	(18)	Joachim Frey
M. leachii	PG50[T]	Australia	1963	Cattle	(14, 32)	Martin Heller
M. leachii	CP291	Portugal	1987	Goat	(33)	Joachim Frey
Mycoplasma bovis	Donetta PG45	USA	1962	Cattle	(34, 35)	Martin Heller
M. bovis	DL589/78	Germany	1978	Calf	(36)	Martin Heller
M. bovis	DL997/79	Germany	1979	Calf	(36)	Martin Heller
M. bovis	DL778/80	Germany	1980	Cattle	This study	Martin Heller
M. bovis	DL097/81	Germany	1981	Calf	(36)	Martin Heller
M. bovis	DL981/84	Germany	1984	Cattle	This study	Martin Heller
M. bovis	DL018/91	Germany	1991	Cattle	(36)	Martin Heller
M. bovis	DL012/92	Germany	1992	Cattle	This study	Martin Heller
M. bovis	11DD0669	Germany	2011	Cattle	This study	Martin Heller

TABLE S1: Strains used in this study

M. bovis	12DD0690	Germany	2012	Cattle	This study	Martin Heller
M. bovis	12DD0691	Germany	2012	Cattle	This study	Martin Heller
M. feriruminatoris	G5847^T	Germany	1993	Alpine Ibex	(37, 38)	Martin Heller
M. feriruminatoris	8756-C13	USA	<1987	Rocky Mountain Goat	(17, 38)	Martin Heller
M. dispar	462/2^T	UK	<1970	Cattle	(39)	Martin Heller
M. buteonis	407/97	Israel	2011	Buzzard	This study	Martin Heller
M. gallisepticum	PG31^T	UK	1977	Poultry	(40)	Martin Heller
M. bovirhinis	PG43^T	UK	1967	Cattle	(41)	Martin Heller
M. bovoculi	M165/69	UK	1972	Cattle	(42)	Martin Heller
M. ovipneumoniae	Y98^T	Australia	<1971	Sheep	(43)	Martin Heller
M. putrefaciens	KS1^T	USA	1955	Goat	(20, 44)	Martin Heller
M. bovigenitalium	PG11^T	UK	1947	Cattle	(45)	Martin Heller
M. californicum	ST-6^T	USA	1981	Cattle	(46)	Martin Heller

TABLE S1: Strains used in this study

M. arginini	G230^T	USA	1968	Mouse	(47)	Martin Heller
M. canadense	275C^T	Canada	1974	Cattle	(48)	Martin Heller
M. verecundum	107	UK	1970	Cattle	(49)	Martin Heller
M. alkalescens	PG51^T	Australia	1961	Cattle	(50)	Martin Heller
M. canis	PG14^T	UK	1951	Dog	(51)	Martin Heller
M. pneumoniae	FH^T	UK	1974	Human	(52)	Martin Heller
Acholeplasma axanthum	S-743^T	UK	1965	Murine tissue culture	(53)	Martin Heller
Acholeplasma laidlawii	PG8^T	UK	1967	Unknown	(54, 55)	Martin Heller
Pastaurella multocida	ATCC 43137^T	Canada	1962	Pig	(56)	Martin Heller

^{T} Type strain

References

1. **Falquet L, Liljander A, Schieck E, Gluecks I, Frey J, Jores J.** 2014. Complete Genome Sequences of Virulent *Mycoplasma capricolum* subsp. *capripneumoniae* Strains F38 and ILRI181. Genome Announc **2**.

2. **Schnee C, Heller M, Jores J, Tomaso H, Neubauer H.** 2011. Assessment of a novel multiplex real-time PCR assay for the detection of the CBPP agent *Mycoplasma mycoides* subsp. *mycoides* SC through experimental infection in cattle. BMC veterinary research **7:**47.
3. **MacOwan KJ, Minette JE.** 1976. A mycoplasma from acute contagious caprine pleuropneumonia in Kenya. Trop Anim Health Prod **8:**91-95.
4. **Pettersson B, Bolske G, Thiaucourt F, Uhlen M, Johansson KE.** 1998. Molecular evolution of *Mycoplasma capricolum* subsp. *capripneumoniae* strains, based on polymorphisms in the 16S rRNA genes. Journal of bacteriology **180:**2350-2358.
5. **Kusiluka LJ, Ojeniyi B, Friis NF, Kokotovic B, Ahrens P.** 2001. Molecular analysis of field strains of *Mycoplasma capricolum* subspecies *capripneumoniae* and *Mycoplasma mycoides* subspecies *mycoides*, small colony type isolated from goats in Tanzania. Vet Microbiol **82:**27-37.
6. **Perreau P, Breard A, Le Goff C.** 1984. Infection expérimentale de la chèvre par les souches de mycoplasme de type F.38 (pleuropneumonie contagieuse caprine). Ann Microbiol (Paris) **135:**119-124.
7. **Bolske G, Johansson KE, Heinonen R, Panvuga PA, Twinamasiko E.** 1995. Contagious caprine pleuropneumonia in Uganda and isolation of *Mycoplasma capricolum* subspecies *capripneumoniae* from goats and sheep. Vet Rec **137:**594.
8. **Jones GE, Wood AR.** 1988. Microbiological and serological studies on caprine pneumonias in Oman. Res Vet Sci **44:**125-131.
9. **Thiaucourt F, Breard A, Lefevre PC, Mebratu GY.** 1992. Contagious caprine pleuropneumonia in Ethiopia. Vet Rec **131:**585.

10. **Lefevre PC, Breard A, Alfarouk I, Buron S.** 1987. *Mycoplasma* species F 38 isolated in Chad. Vet Rec **121:**575-576.

11. **Cordy DR, Adler HE, Yamamoto R.** 1955. A pathogenic pleuropneumonia-like organism from goats. Cornell Vet **45:**50-68.

12. **Perreau P, Breard A.** 1979. La mycoplasmose caprine a *M. capricolum*. Comp Immunol Microbiol Infect Dis **2:**87-97.

13. **Christiansen G, Ernø H.** 1990. RFLP in rRNA genes of *Mycoplasma capricolum*, the caprine F38-like group and the bovine serogroup 7. Zentralblatt für Bakteriologie **20:**479-488.

14. **Thiaucourt F, Lorenzon S, David A, Breard A.** 2000. Phylogeny of the *Mycoplasma mycoides* cluster as shown by sequencing of a putative membrane protein gene. Vet Microbiol **72:**251-268.

15. **de la Fe C, Assuncao P, Rosales RS, Antunes T, Poveda JB.** 2006. Characterisation of protein and antigen variability among *Mycoplasma mycoides* subsp. *mycoides* (LC) and *Mycoplasma agalactiae* field strains by SDS-PAGE and immunoblotting. Vet J **171:**532-538.

16. **Thiaucourt F, Manso-Silvan L, Salah W, Barbe V, Vacherie B, Jacob D, Breton M, Dupuy V, Lomenech AM, Blanchard A, Sirand-Pugnet P.** 2011. *Mycoplasma mycoides*, from "*mycoides* Small Colony" to "*capri*". A microevolutionary perspective. BMC genomics **12:**114.

17. **Manso-Silvan L, Perrier X, Thiaucourt F.** 2007. Phylogeny of the *Mycoplasma mycoides* cluster based on analysis of five conserved protein-coding sequences and possible implications for the taxonomy of the group. Int J Syst Evol Microbiol **57:**2247-2258.

18. **Vilei EM, Korczak BM, Frey J.** 2006. *Mycoplasma mycoides* subsp. *capri* and *Mycoplasma mycoides* subsp. *mycoides* LC can be grouped into a single subspecies. Vet Res **37:**779-790.

19. **Laws L.** 1956. A pleuropneumonia-like organism causing peritonitis in goats. Aust Vet J **32:**326-329.

20. **Tully JG, Barile MF, Edward DG, Theodore TS, Erno H.** 1974. Characterization of some caprine mycoplasmas, with proposals for new species, *Mycoplasma capricolum* and *Mycoplasma putrefaciens*. Journal of general microbiology **85:**102-120.

21. **Dedieu L, Mady V, Lefevre PC.** 1994. Development of a selective polymerase chain reaction assay for the detection of *Mycoplasma mycoides* subsp. *mycoides* S.C. (contagious bovine pleuropneumonia agent). Vet Microbiol **42:**327-339.

22. **Woubit S, Lorenzon S, Peyraud A, Manso-Silvan L, Thiaucourt F.** 2004. A specific PCR for the identification of *Mycoplasma capricolum* subsp. *capripneumoniae*, the causative agent of contagious caprine pleuropneumonia (CCPP). Veterinary microbiology **104:**125-132.

23. **DaMassa AJ, Brooks DL, Adler HE.** 1983. Caprine mycoplasmosis: widespread infection in goats with *Mycoplasma mycoides* subsp *mycoides* (large-colony type). American journal of veterinary research **44:**322-325.

24. **Monnerat MP, Thiaucourt F, Poveda JB, Nicolet J, Frey J.** 1999. Genetic and serological analysis of lipoprotein LppA in *Mycoplasma mycoides* subsp. *mycoides* LC and *Mycoplasma mycoides* subsp. *capri*. Clin Diagn Lab Immunol **6:**224-230.

25. **Cheng X, Nicolet J, Poumarat F, Regalla J, Thiaucourt F, Frey J.** 1995. Insertion element IS1296 in *Mycoplasma mycoides* subsp. *mycoides* small colony identifies a European clonal line distinct from African and Australian strains. Microbiology **141 (Pt 12):**3221-3228.

26. **Westberg J, Persson A, Holmberg A, Goesmann A, Lundeberg J, Johansson KE, Pettersson B, Uhlen M.** 2004. The genome sequence of *Mycoplasma mycoides* subsp. *mycoides* SC type strain PG1^{T}, the causative agent of contagious bovine pleuropneumonia (CBPP). Genome Res **14:**221-227.

27. **March JB, Clark J, Brodlie M.** 2000. Characterization of strains of Mycoplasma mycoides subsp. mycoides small colony type isolated from recent outbreaks of contagious bovine pleuropneumonia in Botswana and Tanzania: evidence for a new biotype. J Clin Microbiol **38:**1419-1425.

28. **Ayling RD, Bisgaard-Frantzen S, March JB, Godinho K, Nicholas RA.** 2005. Assessing the *in vitro* effectiveness of antimicrobials against *Mycoplasma mycoides* subsp. *mycoides* small-colony type to reduce contagious bovine pleuropneumonia infection. Antimicrob Agents Chemother **49:**5162-5165.

29. **Fischer A, Shapiro B, Muriuki C, Heller M, Schnee C, Bongcam-Rudloff E, Vilei EM, Frey J, Jores J.** 2012. The Origin of the '*Mycoplasma mycoides* Cluster' Coincides with Domestication of Ruminants. PloS one **7:**e36150.

30. **Griffin RM.** 1969. Antigenic relationships among strains of *Mycoplasma mycoides* var. *mycoides*, *M. capri* and *M. laidlawii* revealed by complement-fixation tests. J Gen Microbiol **57:**131-142.

31. **Stipkovits L, El-Ebeedy A.** 1977. Biochemical and serological studies of avian mycoplasmas. Zentralblatt fur Veterinarmedizin Reihe B Journal of veterinary medicine Series B **24:**218-230.

32. **Wise KS, Calcutt MJ, Foecking MF, Madupu R, DeBoy RT, Roske K, Hvinden ML, Martin TR, Durkin AS, Glass JI, Methe BA.** 2012. Complete genome sequences of *Mycoplasma leachii* strain PG50T and the pathogenic *Mycoplasma mycoides* subsp. *mycoides* small colony biotype strain Gladysdale. Journal of bacteriology **194:**4448-4449.

33. **Atalaia V, Machado M, Frazao FF.** 1987. Patologia dos pequenos ruminantes infeccões em ovinos e caprinos, originadas pelo micoplasma do grupo 7, Leach (Pg. 50). Rep Trab LNIV **19:**55-60.

34. **Wise KS, Calcutt MJ, Foecking MF, Roske K, Madupu R, Methe BA.** 2011. Complete genome sequence of *Mycoplasma bovis* type strain PG45 (ATCC 25523). Infect Immun **79:**982-983.

35. **Hale HH, Helmboldt CF, Plastridge WN, Stula EF.** 1962. Bovine mastitis caused by a *Mycoplasma* species. Cornell Vet **52:**582-591.

36. **Amram E, Mikula I, Schnee C, Ayling RD, Nicholas RA, Rosales RS, Harrus S, Lysnyansky I.** 2015. 16S rRNA Gene Mutations Associated with Decreased Susceptibility to Tetracycline in *Mycoplasma bovis*. Antimicrob Agents Chemother **59:**796-802.

37. **Fischer A, Santana-Cruz I, Giglio M, Nadendla S, Drabek E, Vilei EM, Frey J, Jores J.** 2013. Genome Sequence of *Mycoplasma feriruminatoris* sp. nov., a Fast-Growing *Mycoplasma* Species. Genome announcements **1:**e00216-00212.

38. **Jores J, Fischer A, Sirand-Pugnet P, Thomann A, Liebler-Tenorio EM, Schnee C, Santana-Cruz I, Heller M, Frey J.** 2013. *Mycoplasma feriruminatoris* sp. nov., a fast growing *Mycoplasma* species isolated from wild *Caprinae*. Syst Appl Microbiol **36:**533-538.

39. **Gourlay RN, Leach RH.** 1970. A new mycoplasma species isolated from pneumonic lungs of calves (Mycoplasma dispar sp. nov.). J Med Microbiol **3:**111-123.

40. **Edward DG, Kanarek AD.** 1960. Organisms of the pleuropneumonia group of avian origin: their classification into species. Ann N Y Acad Sci **79:**696-702.

41. **Leach RH.** 1967. Comparative studies of mycoplasma of bovine origin. Annals of the New York Academy of Sciences **143:**305-316.

42. **Calcutt MJ, Foecking MF.** 2014. Complete Genome Sequence of *Mycoplasma bovoculi* Strain M165/69T (ATCC 29104). Genome Announc **2**.

43. **St George TD, Sullivan ND, Love JA, Horsfall N.** 1971. Experimental transmission of pneumonia in sheep with a mycoplasma isolated from pneumonic sheep lung. Aust Vet J **47:**282-283.

44. **Calcutt MJ, Foecking MF.** 2011. Genome sequence of *Mycoplasma putrefaciens* type strain KS1. Journal of bacteriology **193:**6094.

45. **Edward DG, Freundt EA.** 1956. The classification and nomenclature of organisms of the pleuropneumonia group. J Gen Microbiol **14:**197-207.

46. **Jasper DE, Erno H, Dellinger JD, Christiansen G.** 1981. *Mycoplasma californicum*, a new species from cows. Int J Syst Bacteriol **31:**339-345.

47. **Barile MF, DelGiudice RA, Carski TR, Gibbs CJ, Morris JA.** 1968. Isolation and characterization of *Mycoplasma arginini*: spec. nov. Proc Soc Exp Biol Med **129:**489-494.

48. **Pettersson B, Johansson KE, Uhlen M.** 1994. Sequence analysis of 16S rRNA from mycoplasmas by direct solid-phase DNA sequencing. Appl Environ Microbiol **60:**2456-2461.

49. **Gourlay RN, Leach RH, Howard CJ.** 1974. *Mycoplasma verecundum*, a new species isolated from bovine eyes. J Gen Microbiol **81:**475-484.

50. **Leach RH.** 1973. Further studies on classification of bovine strains of *Mycoplasmatales*, with proposals for new species, *Acholeplasma modicum* and *Mycoplasma alkalescens*. J Gen Microbiol **75:**135–153.

51. **Brown DR, May M, Michaels DL, Barbet AF.** 2012. Genome Annotation of Five *Mycoplasma canis* Strains. Journal of bacteriology **194:**4138-4139.

52. **Bredt W.** 1968. Growth morphology of *Mycoplasma pneumoniae* strain FH on glass surface. Proc Soc Exp Biol Med **128:**338-340.

53. **Tully JG, Razin S.** 1969. Characteristics of a new sterol-nonrequiring *Mycoplasma*. J Bacteriol **98:**970-978.

54. **Lazarev VN, Levitskii SA, Basovskii YI, Chukin MM, Akopian TA, Vereshchagin VV, Kostrjukova ES, Kovaleva GY, Kazanov MD, Malko DB, Vitreschak AG, Sernova NV, Gelfand MS, Demina IA, Serebryakova MV, Galyamina MA, Vtyurin NN, Rogov SI, Alexeev DG, Ladygina VG, Govorun VM.** 2011. Complete genome and proteome of *Acholeplasma laidlawii*. J Bacteriol **193:**4943-4953.

55. **Edward DG, Freundt EA.** 1970. Amended nomenclature for strains related to *Mycoplasma laidlawii*. J Gen Microbiol **62:**1-2.

56. **Davenport KW, Daligault HE, Minogue TD, Bishop-Lilly KA, Bruce DC, Chain PS, Coyne SR, Frey KG, Jaissle J, Koroleva GI, Ladner JT, Lo CC, Palacios GF, Redden CL, Scholz MB, Teshima H, Johnson SL.** 2014. Complete Genome Sequence of Type Strain *Pasteurella multocida* subsp. *multocida* ATCC 43137. Genome Announc **2**.

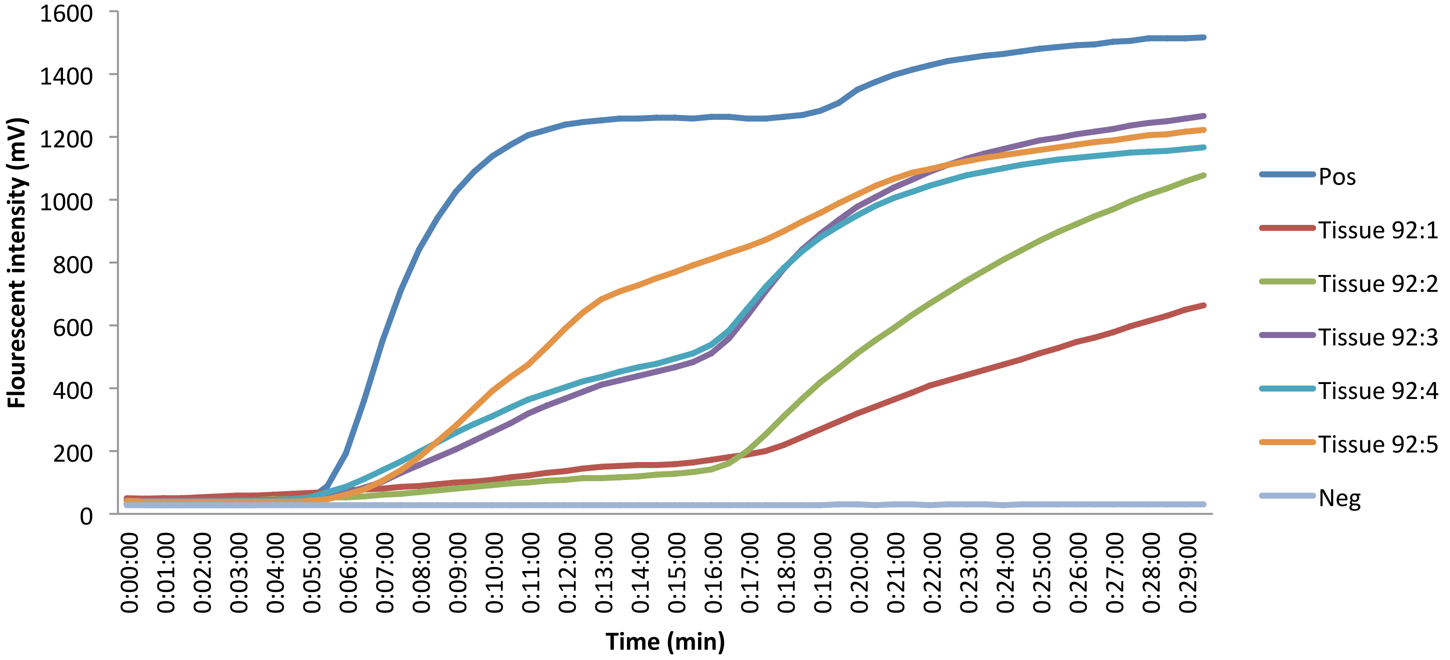

FIGURE S1: Graph depicting the amplification from a tissue sample (Goat 92). Tissue samples (n=5) were homogenized in 0.2M KOH. One micro-liter template was used in the RPA reaction. Positive control; *Mccp* DNA corresponding to 10^7 copies, negative control; 0.2M KOH

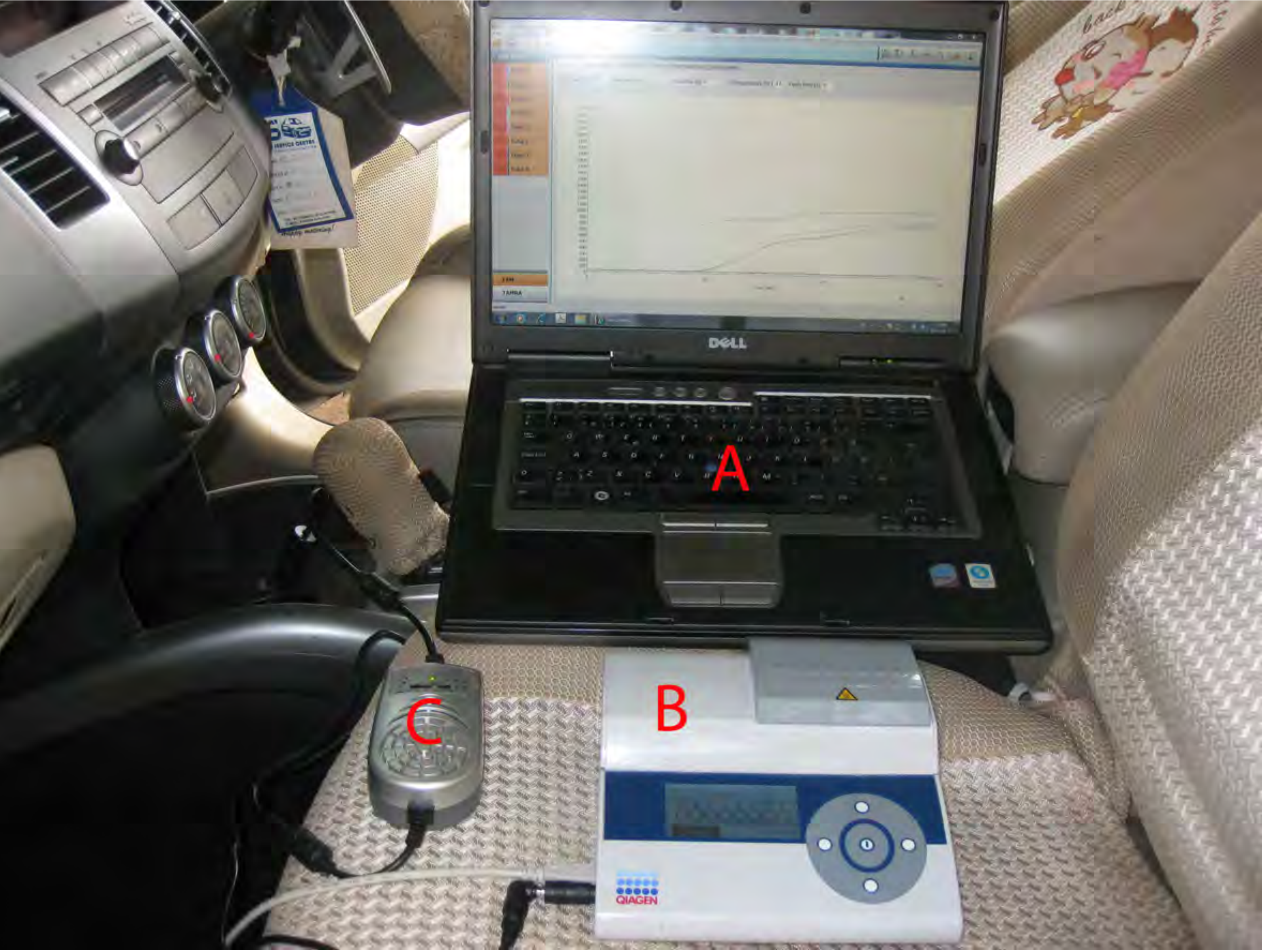

FIGURE S2: P icture depicting the equipment used to run the recombinase polymerase amplification powered by a car battery. A-computer, B-ESEQuant Tube Scanner (Qiagen, Germany), C-adaptor to connect the tube scanner and the computer to the cigarette lighter receptacle in the car.
